# A Device that Models Human Swallowing

**DOI:** 10.1007/s00455-018-09969-2

**Published:** 2019-01-23

**Authors:** M. Stading, M. Q. Waqas, F. Holmberg, J. Wiklund, R. Kotze, O. Ekberg

**Affiliations:** 1 Agrifood and Bioscience Product Design and Perception, RISE Research Institutes of Sweden AB, Göteborg, Sweden; 2 Department of Industrial and Material Sciences, Chalmers University of Technology, Göteborg, Sweden; 3 Diagnostic Centre of Imaging and Functional Medicine, Skåne University Hospital, Lund University, Malmö, Sweden; 4 Animato Konstruktions AB, Domsjö, Sweden

**Keywords:** Pharynx, Deglutition, Deglutition disorders, In vitro, Shear rate, Rheology, Manometry

## Abstract

The pharynx is critical for correct swallowing, facilitating the transport of both air and food transport in a highly coordinated manner, and aberrant co-ordination causes swallowing disorders (dysphagia). In this work, an in vitro model of swallowing was designed to investigate the role of rheology in swallowing and for use as a pre-clinical tool for simulation of different routes to dysphagia. The model is based on the geometry of the human pharynx. Manometry is used for pressure measurements and ultrasonic analysis is performed to analyze the flow profiles and determine shear rate in the bolus, the latter being vital information largely missing in literature. In the fully automated model, bolus injection, epiglottis/nasopharynx movement, and ultrasound transducer positioning can be controlled. Simulation of closing of the airways and nasal cavity is modulated by the software, as is a clamping valve that simulates the upper esophageal sphincter. The actions can be timed and valves opened to different degrees, resembling pathologic swallowing conditions. To validate measurements of the velocity profile and manometry, continuous and bolus flow was performed. The respective velocity profiles demonstrated the accuracy and validity of the flow characterization necessary for determining bolus flow. A maximum bolus shear rate of 80 s^−1^ was noted for syrup-consistency fluids. Similarly, the manometry data acquired compared very well with clinical studies.

## Introduction

Swallowing, which is the final stage of oral processing, ensures smooth transport of the orally processed food towards the stomach for further digestion [1]. Human swallowing is an involuntary action that takes place about 1000 times a day [2]. Pharyngeal swallowing is important to study since the pharynx is partly shared by the airways and food swallowing tract [3]. Misdirection of the bolus at this stage means that the bolus enters the airways, resulting in aspiration and possibly, pneumonia. Swallowing disorders (dysphagia) are a growing concern and it is estimated that dysphagia affects roughly 8% of the world population [4]. The physiological responses of people who are suffering from dysphagia caused by neurological conditions or age-related impairment are insufficient to handle the rapid flow of foods or liquids through the oropharynx. Therefore, thickeners, which are typically gum- or starch-based, are added to the food to slow down the flow of the bolus. Starch-based thickener swells upon hydration while gum-based thickeners form network thereby holding water and increasing the viscosity. A thickener, whether gum- or starch-based shear thins during flow, i.e., the perception of thickness decreases with increasing speed of deformation [5]. This necessitates accurate information of shear rate during swallowing. European Society for Swallowing Disorders (ESSD) in its recently published White Paper, has stressed the importance of rheological parameters such as shear rate, non-Newtonian fluids properties, yield stress, elasticity, and density [6]. To test these parameters in humans is not only cumbersome but it also possesses ethical issues. For example in our earlier work, aimed at relating elasticity and safe swallowing, it was noticed that even though high elasticity in liquids has an effect on safe swallowing, the results were not statistically significant unless a large number of patients were evaluated and variability among subjects was kept small [7].

The assessment tool used by clinicians is either manometry or video-fluoroscopy [8, 9]. Manometry is clinically vital but technically hard to perform [10]. While executing manometry, distinction between hydrodynamic pressure, bolus pressure as it touches transducer and contact pressure, measured pressure as pharyngeal wall touches the transducer must be made [10]. Furthermore, manometry is based on the insertion of a probe into the patient’s pharynx, which obstructs the bolus flow [8, 9] and causes discomfort. During video-fluoroscopic analysis, the swallowing of fluids is monitored using X-ray imaging and the entire swallowing process is recorded, therefore enabling the examiner to follow the swallowing sequence frame by frame [11]. However, video-fluoroscopy necessitates the use of radio-opaque contrast media that has been to shown to alter the rheology of the bolus [12].

To analyze different parameters suggested in the White Paper by ESSD, we propose bolus flow measurement using ultrasound velocity profiling (UVP) technique, which detects the movement of suspended particles/bubbles in a flowing liquid using Doppler echography. The technique, which is non-invasive and can measure velocity profiles in real time, can be applied to measure the velocity profile of the bolus and thereby determine accurate shear rate during swallowing. The UVP technique is described in detail elsewhere [13].

The swallowing process has been simulated in vitro by Mackley et al. [14] and Noh et al. [15] previously (existing models are reviewed in [16]). The model presented by Mackley et al. named “The Cambridge throat” does not simulate the epiglottis movement during bolus flow hence mimicking only severe dysphagia. The Cambridge throat did not report the shear rate and manometry information that is vital to study the influence of rheology with respect to swallowing. Nevertheless, the Cambridge throat presents a good starting point for in vitro simulation of swallowing process. The in vitro model presented by Noh et al. used video-fluoroscopy to follow the bolus flow thus mimicking in vivo equivalent of swallowing analysis. The model published by Noh et al. lacks epiglottis structure and does not report shear rate and manometry information that dysphagia community is most interested in, as mentioned earlier. Both these models conclude that a thickened bolus travels slower in the oropharynx and too thick consistency bolus leaves post-swallow residues as also noticed in clinical studies [4]. Moreover, several theoretical and simulation studies have been performed on the swallowing process [17–21]. Simulation studies normally assume highly idealized conditions, such as uniform bolus geometry and Newtonian fluids. Considering that most boluses, especially the ones thickened for dysphagia are shear thinning and often elastic, means that at least non-Newtonian fluids have to be considered. Furthermore, the complex geometry and peristaltic type of motion exposing the bolus to complex sequence of shear and extension deformation makes mathematical modeling even more challenging [16]. For detailed description of existing in vitro swallowing models, the readers are advised to the book chapter [22]. In our opinion, an in vitro simulator that performs in vivo type of analysis without interfering with the human body provides the best compromise between the two extremes of modeling and clinical studies.

Therefore, the aim of the present work was to design an in vitro device that mimics human swallowing, and that could be used to study the rheological parameters suggested in the White Paper by ESSD. Furthermore, the device should allow studies of the flow properties of the bolus and to simulate both healthy swallowing and various swallowing disorders. In this work, we presented for the first time the shear rate distribution during bolus swallowing using non-invasive UVP technique. Additionally, the physiologically realistic geometry was utilized to perform in vitro manometry.

## The In Vitro Model Design and Validation

This section presents briefly presents the in vitro model, later on validation of the results with the device is presented.

### Design Concept of the Model

The design of the in vitro model takes into account:

- the actual human geometry;
- simulation of the physiologic processes: closing of the vocal chords, the upper esophageal sphincter (UES) and the epiglottis, and opening to the nasopharyngeal channel;
- monitoring of the bolus velocity profile during swallowing;
- pressure measurements at different locations of interest, i.e., at the entrance to the pharynx, mid-pharynx, at the UES, and in the nasopharynx;
- manometry at the entrance of pharynx, mid-pharynx and UES, mid-pharynx and nasopharynx;
- a transparent pharyngeal channel for visual observations; and
- temperature control.

**Fig. 1:**
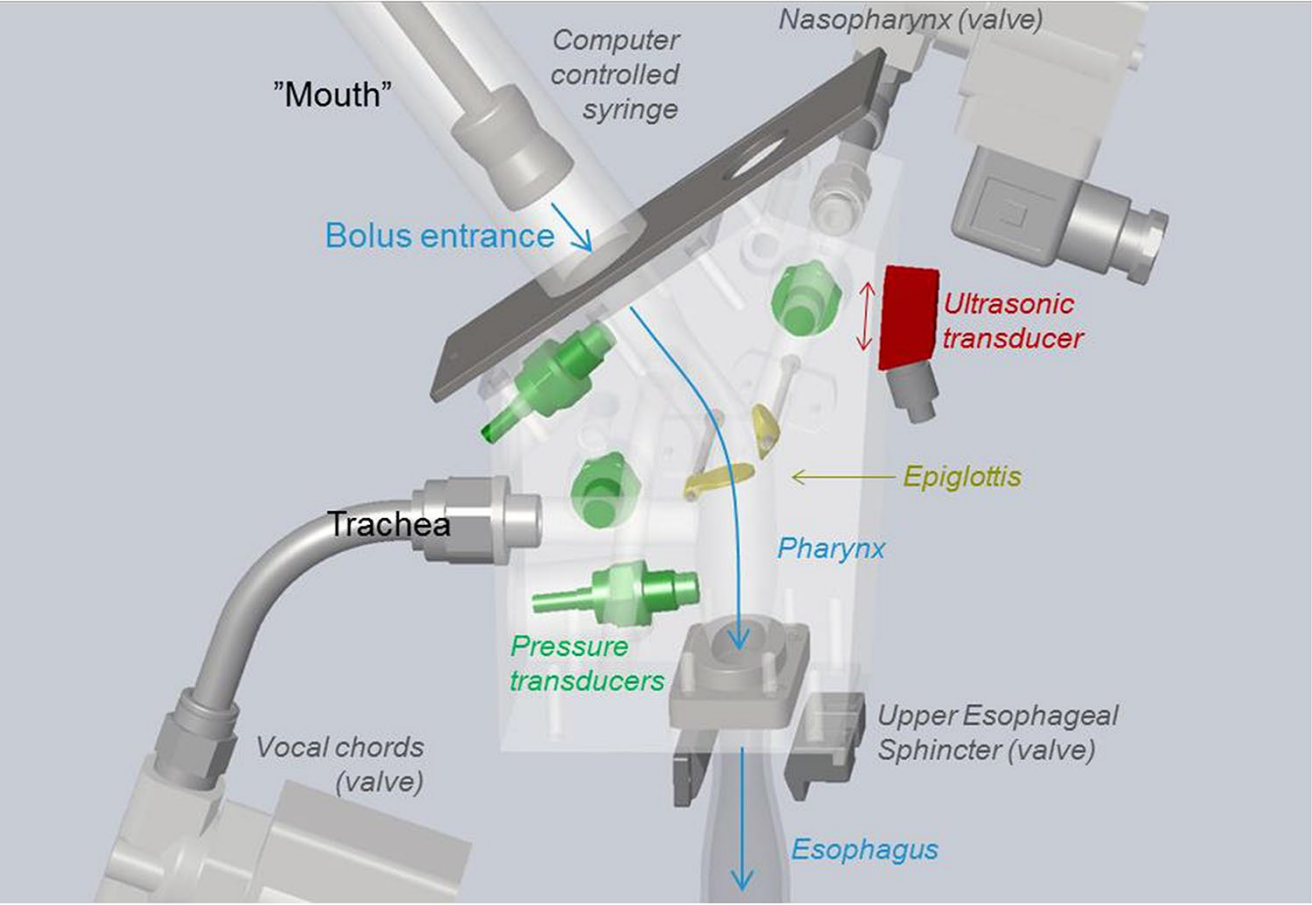
Schematic of the in vitro Gothenburg Throat model. The oral phase is mimicked by a syringe that delivers a bolus into the pharyngeal channel. The transparent block hosts the pharyngeal channel, the opening to the trachea, and the opening to the nasopharynx. The bottom of the pharyngeal channel connects to the esophagus. Pressure transducers are shown in green; movable “lids” (the epiglottis and entrance to the nasopharynx) are shown in yellow; and the ultrasonic transducer that monitors the bolus velocity profile is indicated in red

An overview of the in vitro model is shown in Fig. 1, and the individual components are described in detail below.

#### Oral Phase

In the model, the oral phase is mimicked using a syringe that can deliver a bolus of fixed volume and at a fixed speed. This simulates the thrusting actions of the tongue and gum when swallowing is initiated. Model bolus volumes fall in the range of 5–30 ml (physiologically, it is typically 20 ml). Model bolus speed at the pharynx is in the range ~ 0.5–1.0 m/s for healthy individuals. The bolus is loaded from a tank, enabling automated repeated measurements. A sliding valve is located after the syringe, before the entrance to the pharynx, to avoid gravity-induced flow before active bolus delivery.

#### Pharyngeal Physiology and Epiglottal Action

Pharyngeal swallowing is involuntary, being initiated with the trajectory of the hyoid bone, which moves both upward and forward, thereby facilitating the expansion of the pharynx. The swallowing center lying in the brain stem responds to the incoming information by sending appropriate signals to the related structures through the cranial nerves [23]. As a result, the epiglottis (one of the structures) tilts down to cover the airways, thereby partially arresting respiration as the bolus travels towards the esophagus.

**Fig. 2:**
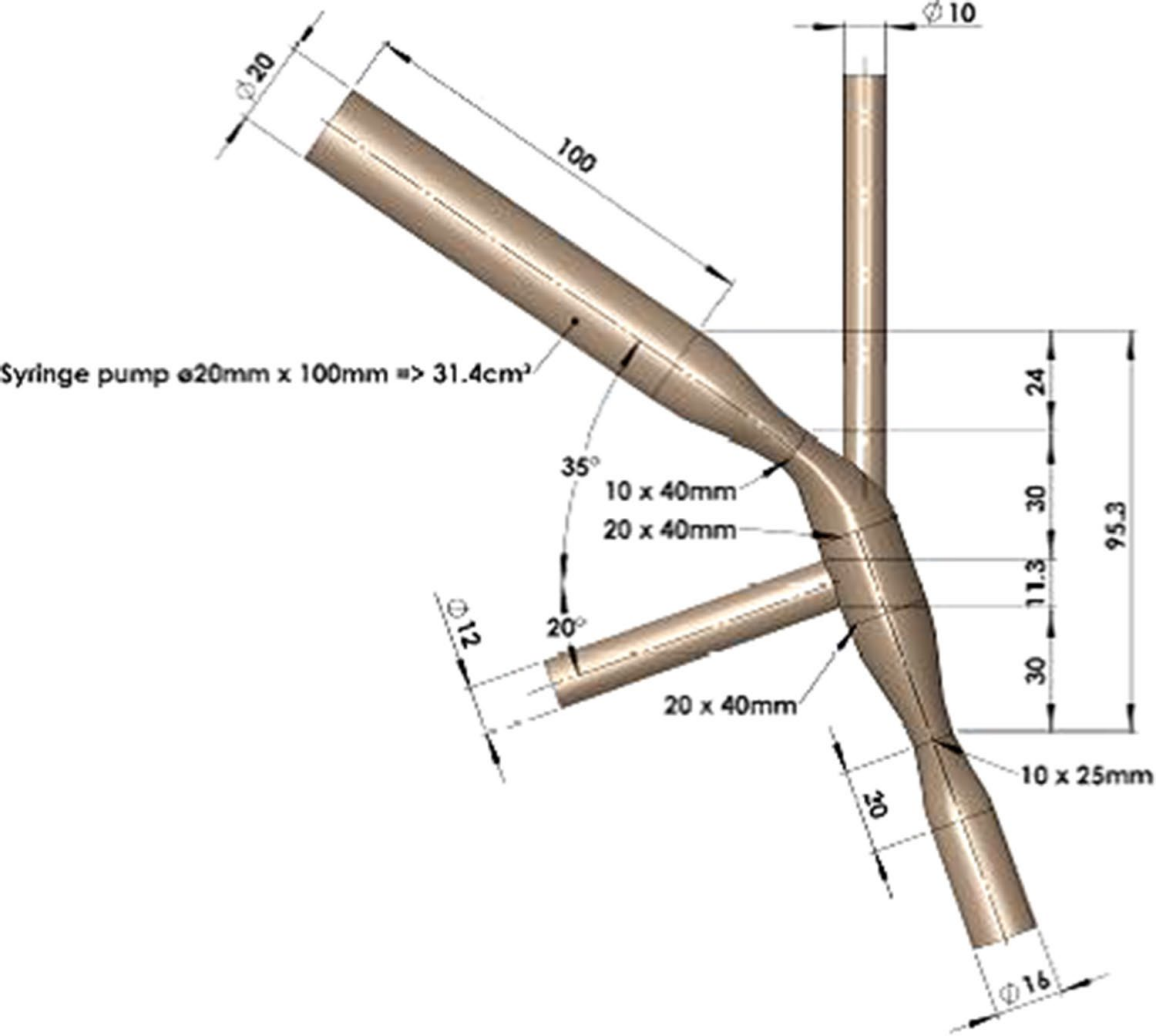
Basic design and dimensions of the structure of the model pharynx corresponding to the human distended form

During pharyngeal swallowing, the geometry of the passage changes and acquires an elliptical shape as the bolus passes through it [10]. Thus, a perfect in vitro model should mimic the changing geometry, which was not possible with the current setup, as a transparent and ultrasound-transmissible model was required for performing the measurements. Hence the pharynx was constructed in the distended form, the shape that pharynx acquires as it hosts the bolus. Thus, the pharynx in the model is elliptical (Fig. 2), with a length of 6.3 cm, as taken from a previous study [24], width of 2.8–3.0 cm at its widest point [20], and a circular entrance area of 314 mm^2^ (diameter = 20 mm). The initial circular area is a compromise so as to be able to use a syringe with circular cross-section for bolus delivery. The material chosen was Accura^®^ ClearVue, due to its suitability for usage in medical models, transparency for fluid flow visualization, and liquid resistance. This material can also be 3D printed, as was the case for the Gothenburg Throat.

The epiglottis is a leaf-like organ with length of approximately 11.3 mm, maximum diameter of 12.5 mm, and average thickness of 1.87 mm [25]. The epiglottis in the model (Fig. 2) can rotate to an angle of 60° during 0.2 s, an angular velocity that is sufficient to host closure when water is passing through, reported to take 0.22 ± 0.09 s [26]. The stepper motor that supplies the motion for the epiglottis and nasopharynx was obtained from Faulhaber GmbH (Schönaich, Germany), and used because of its suitability for small-sized objects.

#### The Upper Esophageal Sphincter

**Fig. 3:**
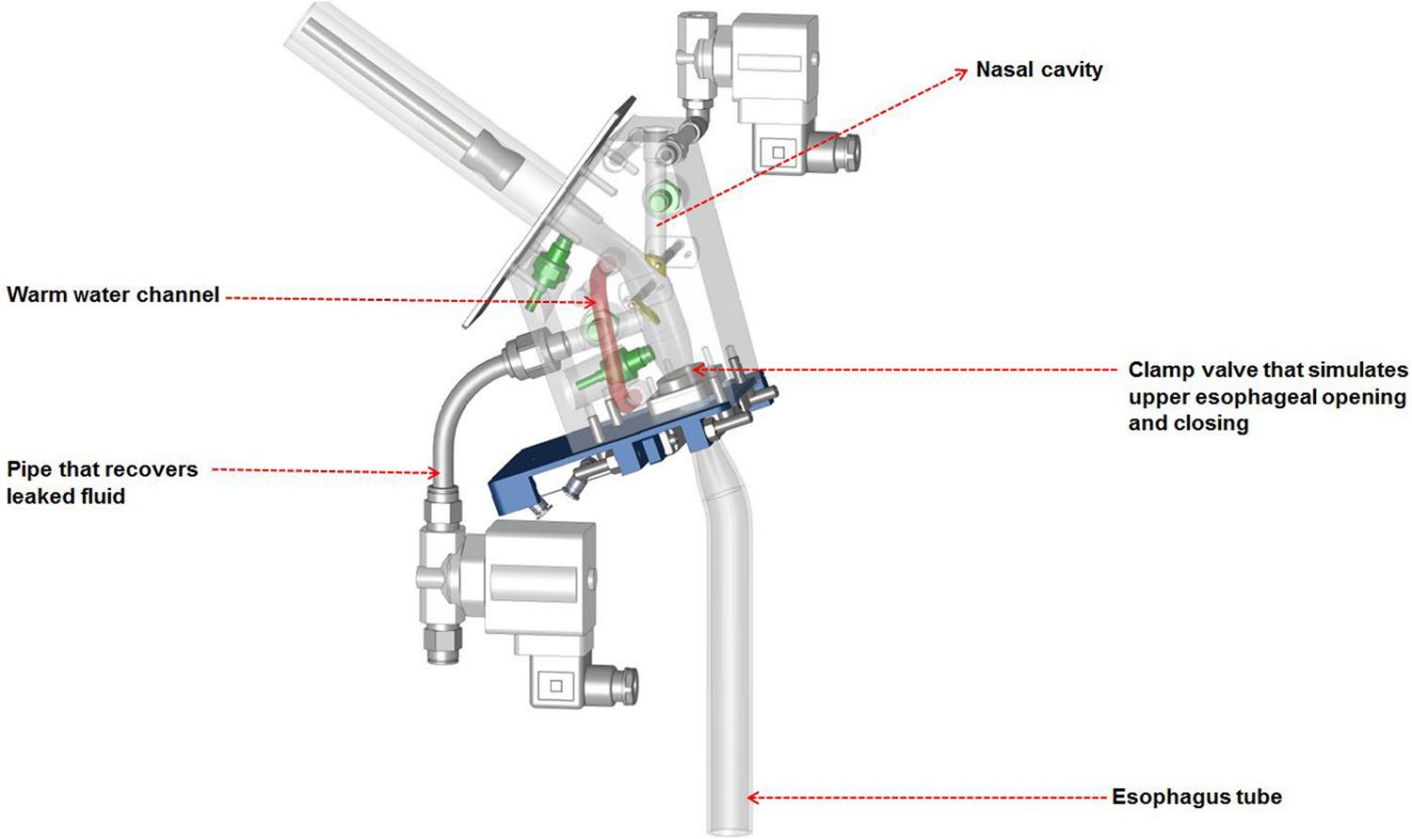
Schematic of the modeled epiglottis (yellow), nasal cavity, upper esophageal sphincter (circled), opening/closing function of the UES controlled by a clamp valve (blue), pressure transducers (green), the warm water channel (in red), and the part of the trachea that recovers the leaked fluid when abnormal swallowing is simulated

The UES, which lies at the junction of the pharynx and the esophagus [8], ensures a smooth transition of materials from the pharynx to the esophagus, and in the normal state is closed. The UES must open at the right time for safe swallowing to occur, otherwise aspiration of the bolus can occur [27]. The UES has a length of 2–4 cm and a diameter of 0.95 ± 0.15 cm. During swallowing, the UES opens for 415 ± 66 ms [8]. The UES in our model is 4.3 cm in length and has an elliptical entrance. The model UES can be opened and closed using a pinch valve, thereby simulating the action of the UES during swallowing (Fig. 3). The esophageal structure is modeled by a transparent rubber tube that is 20 cm in length and 1.7 cm in diameter, mimicking the human physiology [23].

#### The Nasal Cavity

The consistency of the bolus has a significant effect on aroma release [28]. The nasal cavity is included in the model using a channel with circular cross-sectional area of 78 mm^2^ (diameter = 10 mm) and length of 40 mm. The cavity can be opened and closed by a valve (Fig. 3), while a revolving lid covers the entrance of the opening to the nasal channel to ensure a smooth inner geometry of the pharynx when the bolus passes through. The lid is controlled by a motor (Faulhaber GmbH) and can be opened and closed to different degrees. This nasal function has been added to enable studies of the breathing–swallowing relationship.

#### The Trachea

The human trachea is a tube with a wall that contains cartilage and connects the pharyngeal cavity and the respiratory system. The trachea is 57.13 ± 7.32 mm in length with a circular shape of diameter 12 mm [29]. During pharyngeal swallowing, the trachea lifts upward and forward to distend the pharynx and to receive and transport the bolus safely towards the esophagus without allowing entrance into the trachea. During bolus transport, the trachea is protected by contraction of the laryngeal muscles and movement of the epiglottis [23].

During normal swallowing, the airways are closed completely by the vocal chords. In the model, a valve shuts off the airflow through the trachea (Fig. 3). The internal diameter of the trachea in the model is 12.3 mm. A pressure transducer is installed in the trachea to measure the pressure during swallowing.

#### Temperature Control

People who suffer from dysphagia often have a slow oral phase [7]. Therefore, consideration of body temperature is even more imperative. To mimic an accurate body temperature, channels for circulating temperature-controlled water are installed in the model and in the syringe delivering the bolus. A temperature transducer is installed to monitor the temperature inside the model, as shown in Fig. 3.

### Components

The components used to construct the Gothenburg Throat model are listed in Table 1.

**Table 1:** Components used in the Gothenburg Throat model

Components	Manufacturer	Cities	Countries
Pneumatic syringe	Aventics	Eger	Hungary
Linear unit to mount the ultrasound transducer	THK	Tokyo	Japan
Ultrasound transducer	Incipientus^®^	Gothenburg	Sweden
Pressure transducers	TE Connectivity	Paris	France
Pico oscilloscope for pressure data acquisition	Pico Technology	Cambridgeshire	England
Epiglottis	Prototal	Jönköping	Sweden
Plastic for the pharynx (Accura^®^ ClearVue™)	3DSYSTEMS^®^	Rock Hill	USA
Pressure regulators	Aventics	Eger	Hungary
Slide valve	NYBERGS	Borlänge	Sweden
UES body	NYBERGS	Matfors	Sweden
UES design	NYBERGS	Matfors	Sweden
Circulating heat jacket	NYBERGS	Matfors	Sweden
Micromotor to rotate the epiglottis and nasopharynx	Faulhaber	Schönaich	Germany
Esophagus tube	Esska	Arvika	Sweden

### Software

**Fig. 4:**
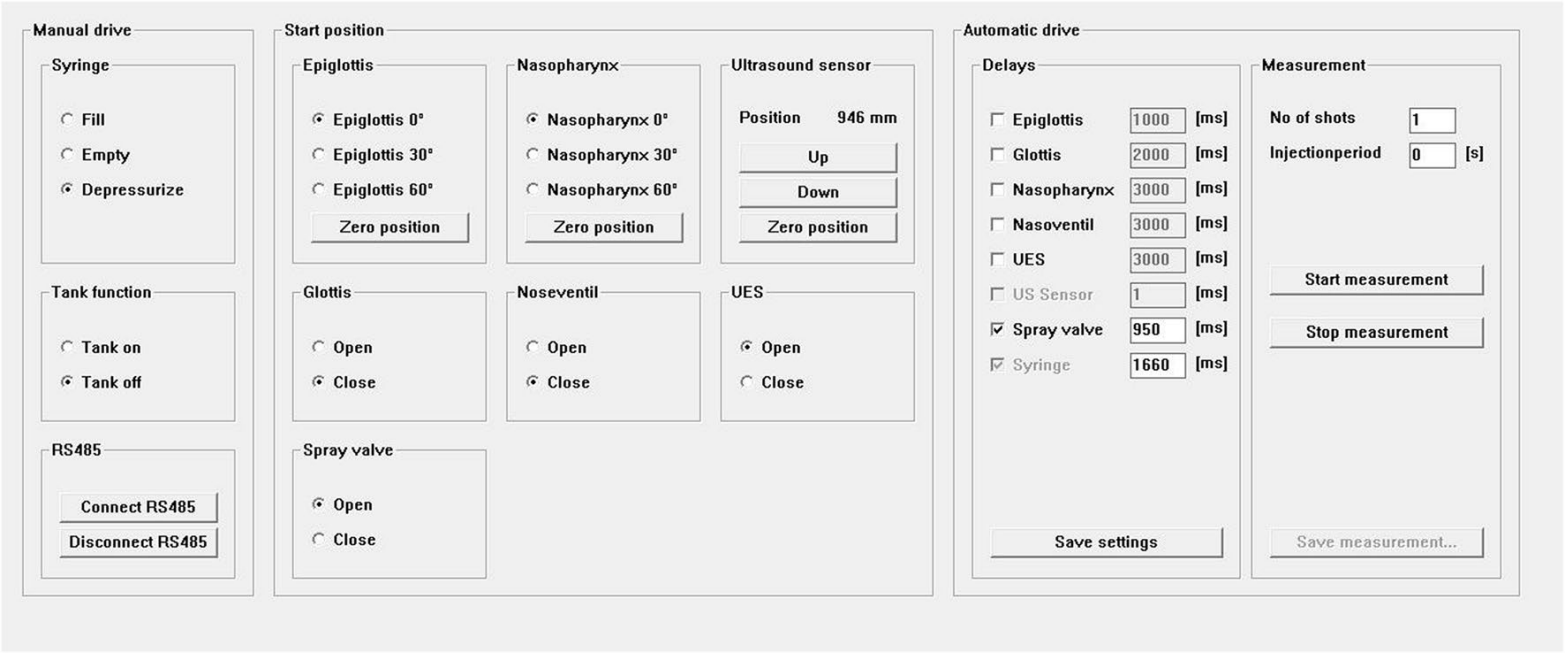
User interface of the software used to control operation of the model

The software used to regulate the syringe, valves, and lids was developed in-house using C language (Fig. 4), and compilation for Windows was performed by the compiler Pelles C (Smorgasbordet, Stockholm, Sweden). The interval between bolus injections can be set in seconds. Measurements can be stopped at any time during the flow. Opening and closing of the structures (epiglottis, glottis, nasopharynx, UES, and slide valve) can be timed to reflect different physiological conditions. The software controls the position of the ultrasound transducer linearly from a distance of 7.5 mm from the dorsal side using a brushless DC motor enabling the velocity profile measurement at different locations. The software performs all the necessary control functions, including (1) regulating the motions of the structures of the nasopharynx, trachea, and UES, (2) controlling the valves for the trachea and nasopharynx, and (3) controlling pneumatic operation of the syringe and slide valve responsible for bolus injection. Moreover, cleaning during batches is controlled by the software in terms of filling, emptying, and regulating the continuous flow from the tank.

### In Vitro Manometry

#### Pressure Transducers

Pressure transducers from TE Connectivity (Paris, France) are installed in the model at four locations, in the pharyngeal entrance; mid-pharynx; and nasal cavity. The transducers are designed to measure dynamic pressures in small and complex geometries, such as those in our in vitro model. The pressure transducer can measure up to ± 48 kPa with the given electronic settings. These values (> 40 kPa) are sufficient to mimic most of the pressure values reported during human swallowing, which typically are around 48 kPa for men and 42 kPa for women [1].

#### Manometry Measurement in Continuous and Bolus Flow

Pressure measurements are performed using an oscilloscope from Pico Technology (Cambridgeshire, England). The linear range of the voltage can be set in the software to correspond to the pressure (in kPa) as an output unit. The electronics performs band pass filtering (0.1 Hz to 1 kHz) to compare the bolus pressure to atmospheric pressure and to filter out high-frequency noise. The software performs continuous data acquisition separately from the control software upon detecting physical activity inside the model. The pressure transducers were controlled against a digital reference pressure transducer, DPI 705 (Amtele Engineering AB, Stockholm, Sweden) for air and was found to be accurate within < 6% in the pressure range of 1–48 kPa.

## Materials and Methods

### Materials

Commercial rapeseed oil was used as a model Newtonian fluid (ICA-Maxi, Sweden) and was compared to the shear-thinning fluid Fresubin Clear (Fresenius-Kabi GmbH, Bad Homburg, Germany), which is a thickener used to manage dysphagia. Rapeseed oil was used as is, whereas Fresubin Clear was mixed to syrup consistency by adding 3.91 g of the powder to 100 ml of water.

### Methods

The shear viscosity of the samples was determined with the ARES-G2 rheometer (TA Instruments, New Castle, DE, USA), using a cone-plate geometry with plate diameter of 40 mm and cone angle of 0.04 rad. Sample density was measured with the Densito 30PX density meter (Mettler Toledo AB, Stockholm, Sweden).

#### Flow Visualization and Verification Using Ultrasonics

Flow visualization was performed using the UVP technique. In this work, a commercial UVP system that included electronics, software, and transducers was used (Incipientus™ Ultrasound Flow Technologies AB, Gothenburg, Sweden).

### Experimental Loop for UVP and Manometry in a Continuous Flow

To validate that measurements can be performed in the given geometry with ultrasound and pressure transducers, initially the fluids were continuously pumped from a tank using a rotatory lobe pump (Sterilobe SLAS; Johnsons Pumps, Lanarkshire, UK) operated at two different speeds, corresponding to two different flow rates (Tables 2, 3).

**Table 2:** Percent difference between flow rates measured with the gravimetric method and velocity profile integration (UVP) methods, shear rate at 50 s^−1^, and the physical properties of the test fluids

Fluids	Gravimetric (l/min)	UVP (l/min)	% Deviation gravimetric and UVP methods	% Deviation gravimetric method and Eq. 1$\left( {Q = \frac{\pi }{2}vab} \right)$	Viscosity at shear rate 50 s^−1^
Rapeseed oil	0.97	0.89	8.14	1.15	0.063
	2.36	2.21	6.1	6.4	
Fresubin Clear	1.14	0.98	13.65	43.78	0.65
	2.78	2.06	25.8	38.45	

All the numerical values in the table are mean of at least three measurements with standard deviation < 5%. Comparison of the UVP and Eq. 1 with gravimetric method is performed to demonstrate the accuracy of results both through the applied technique (UVP) and mathematically

**Table 3:** In vitro shear rate and corresponding maximum velocities recorded

Bolus injection numbers	Velocity (m/s)	Shear rate (s^−1^)
1	0.22	124
2	0.161	78.83
3	0.162	67.6
4	0.144	71
Average	0.170	85.36
Standard deviation	0.031	39.76

The entire model was filled with the fluid, and the valves on the trachea and nasal cavity were closed. The ultrasound transducer was attached using a specially designed holder to ensure firm contact between the transducer face and the model. For optimal transmission of the acoustic waves, rapeseed oil was introduced between the transducer and the model pharynx block as coupling media. Several commercially available acoustic media were tested before and rapeseed oil was finally selected due to better matching of acoustic impedance with the given material used in the model pharynx. The temperature of the fluid was strictly monitored during the experiments within 20.5–22.0 °C.

Validation of the manometry was performed during continuous flow using the pressure drop between the lower, mid-pharynx pressure transducer (see Fig. 1), and the outlet (atmospheric pressure), and comparing this to the calculated pressure drop using the Hagen–Poiseuille law and the gravimetrically measured mass flow rate. In order to eliminate geometrical effects, the pressure drop is presented in a simplified form as the ratio of the pressure at a high-flow rate (*P*_H_) to the pressure at a low-flow rate (*P*_L_): $\frac{{(P_{\text{H}} - P_{0} )}}{{(P_{\text{L}} - P_{0} )}}.$

Pump speeds corresponding to the flow rates reported in Table 2, where *P*_0_ is the atmospheric pressure at the fluid exit point, were applied.

Similarly, the flow rates are expressed as the ratio of the high-flow rate ($\dot{Q}_{\text{H}}$) to the low-flow rate ($\dot{Q}_{\text{L}}$): $\left( {\frac{{\dot{Q}_{\text{H}} }}{{\dot{Q}_{\text{L}} }}} \right)^{n}$ with *n* representing the power law coefficient (*n* = 1 for the Newtonian oil, and *n* < 1 for the shear-thinning thickener solution). According to the Hagen–Poiseuille law, the changes in pressure drop and flow rates should be equal as in Eq. 1:

1 $$\frac{{(P_{\text{H}} - P_{0} )}}{{(P_{\text{L}} - P_{0} )}} = \left( {\frac{{\dot{Q}_{\text{H}} }}{{\dot{Q}_{\text{L}} }}} \right)^{n} .$$

The data were acquired when the pressure values reached a steady state, as monitored by the PicoScope software (Pico Technology, Cambridgeshire, England). The measurements were repeated three times and the standard deviation from the mean value did not exceed 2.5%.

### Methodology for UVP Measurements

The ultrasonic beam passes through the polycarbonate material of the model into the actual fluid flow (Fig. 1) which means that it will be refracted at each interface. The Doppler angle, i.e., the angle between the fluid flow and ultrasonic beam, inside the model cavity was measured using a reference 90° ultrasound beam. The two values were utilized to determine the angle inside the model pharynx for each test fluid.

An average of 128 velocity profiles was used to determine the volume flow rate and shear rate. A Doppler angle of 60° and ellipse short-axis radius 8.4 mm was determined, while the sound velocities in rapeseed and Fresubin Clear were 1443.6 m/s and 1560 m/s, respectively. The base frequency of the non-invasive ultrasound transducer was 5 MHz, and 5 cycles/pulse were used for velocity profile measurements.

#### Shear Rate Calculation from the Velocity Profile

Shear rate distribution (Eq. 2) from the wall to the center $\dot{\gamma }$ is calculated from the gradient of velocity profiles (*v*), recorded with the UVP device as

2 $$\dot{\gamma } = - \frac{{{\text{d}}v(r)}}{{{\text{d}}r}}.$$

To capture the velocity gradient, a second-order polynomial was fitted to the velocity profiles recorded with the UVP. The highest shear rate is the one calculated from the velocity gradient at the wall of the model pharynx.

This method of shear rate measurement is well established and documented in many published articles as [13, 30–32]. The shear rate presented is a measure on four bolus injections in the model cavity. Each data set representing an average of 128 velocity profiles is processed individually with UVP software.

### Reference Measurement

The total volumetric flow rate through a cylinder with elliptical cross-section for a Newtonian fluid is given by Eq. 3 as found in literature [33]

3 $$Q = \frac{\pi }{2}v a b,$$

where *v* is the maximum velocity at the center of the tube, and *a* and *b* are the axes of the ellipse, in this case 8.4 mm and 18.2 mm, respectively.

To compute the volumetric flow rate ($\dot{Q}$) through a cross-section of the pharynx, the cross-section is first broken up into *n* segments, each with area *A*_*n*_. The velocity in each segment is denoted *V*_*n*_.

Then, assuming negligible secondary flow, the volumetric flow rate can be calculated using Eq. 4:

4 $$Q = \sum\limits_{n = 1}^{N} {\left( {V_{n} A_{n} } \right)} .$$

The conventional Bucket and StopWatch (BSW) method was used as a reference measurement. Thus, the mass flow rate was measured as a function of time by filling a bucket with the test fluid while recording time using a stopwatch. The mass flow rate was then converted to the volumetric flow rate ($\dot{V}$) using the relation $\dot{V} = \frac{{\dot{m}}}{\rho },$ where *m* is the mass and *ρ* is the density of the test fluid. These parameters were measured with a high-precision electronic scale (PG4002-S; Mettler Toledo AB, Stockholm, Sweden) and a digital density meter. This procedure was repeated at least five times for each sample with two flow rates of the pump. The average value was used as the reference and compared with the flow rate determined using UVP (Eq. 4) and the error difference percentage was calculated.

### In Vitro Bolus Injection Procedure

Final bolus injection settings were chosen based on initial pre-experiment. The main criterion was to ensure a smooth flow of a bolus of defined volume (15 ml in this case) released by the syringe. The UES was closed after 3 s after the bolus departed from the pharynx. The final volume of liquid considered for analysis is the one collected after the UES closing the esophagus pipe and that volume varied 1.2 ml around the mean value of 15 ml.

## Validation of the Results

Measurements performed to validate the UVP and manometry results are discussed in the following sections.

### Ultrasound Velocimetry Profiling in Continuous Flow

**Fig. 5:**
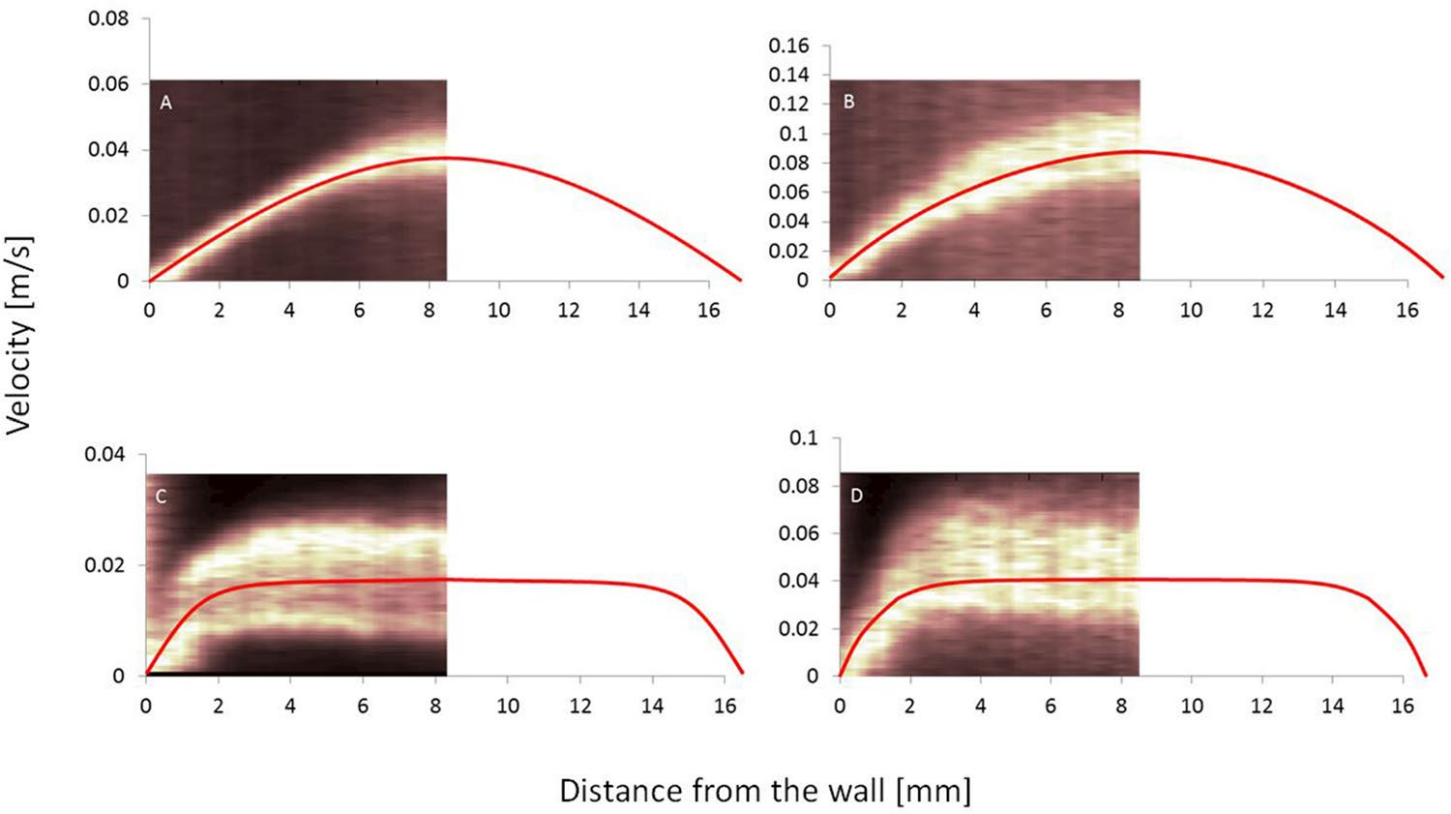
**a** and **b** Velocity profiles (m/s) of rapeseed oil at flow rates of 0.97 and 2.36 l/min. **c** and **d** Velocity profiles of Fresubin Clear at flow rates of 1.14 and 2.78 l/min. The red line superimposed in individual figure shows an average of the 128 velocity profiles recorded. Left panel in every individual figure shows the power spectra measured by the UVP device up to the center of the model pharynx. In the right panel, the red line is extended towards the remaining half of the modern pharynx to show the fitted velocity profiles in m/s

The results reveal that UVP accurately performs flow visualization in the in vitro model. The Doppler spectra and average velocity profiles (red) measured at two different flow rates in rapeseed oil (0.97 and 2.36 l/min) and Fresubin Clear (1.14 and 2.78 l/min) in the in vitro model are presented in Fig. 5. A typical Newtonian velocity profile was acquired for rapeseed oil (Fig. 5a, b), whereas Fresubin Clear demonstrated a shear-thinning, plug-flow profile at the two flow rates (Fig. 5c, d).

The velocity profile measurements revealed a clear distinction between two rheologically different fluids. Rapeseed oil, which is a simple Newtonian fluid, yielded a parabolic velocity profile across the given model pharynx. Fresubin Clear, which is a more complex fluid that is composed of many ingredients (modified starch, maltodextrin, xanthan gum, and modified cellulose) yielded a plug-flow profile. Of these ingredients, cellulose and starch in modified form have surface-active properties [34]. This results in foam formation, which can arrest and immobilize particles of smaller size such as air bubbles and thereby promote yield stress. Yield stress in a fluid demonstrates the strength of the coherent network structure. This is discussed in much detail in our previous article [35], in which Fresubin Clear was the fluid that recorded the highest yield stress.

Furthermore, air causes attenuation of the ultrasound energy resulting in weak echo signal. Moreover, the presence of air bubbles in a fluid results in individual fluid layers traveling at different speeds, this is depicted in broadening of the spectrum as more Doppler velocities are measured (see Fig. 5c, d). Hence when comparing the two flow rates, larger error differences were observed.

To verify the accuracy of the measurements, the gravimetric BSW method was used as a reference. Table 2 shows the flow rates acquired, the velocity profile integration (UVP), and the percentage difference between the two methods. The UVP method yielded good agreement, with only 6–8% difference noted for rapeseed oil at the measured flow rates. However, the difference between the two methods increased up to 25.8% in the more complex fluid, Fresubin Clear, at the maximum flow rate of 2.78 l/min due to excessive accumulation of air.

For the method presented in the literature [33], Eq. 1 takes into consideration only the maximum velocity inside the ellipse and is only applicable to Newtonian fluids. Therefore, the method in the literature gives good agreement with the results for rapeseed oil (maximum of 6.4% difference). However, the deviation increases up to 44% when comparing with a non-Newtonian fluid, Fresubin Clear (see Table 3). The good agreement between UVP and Eq. 1 (< 10% difference) further verifies the method, so it can be concluded that the methodology used is suitable for the performance of flow measurements inside the model pharynx on a bolus.

### Ultrasound Velocimetry Profiling Measured on Boluses

To demonstrate ultrasonic measurement on actual bolus flow, Table 3 shows the velocity profiles and the corresponding wall shear rate calculated. Four bolus injections were performed, where the velocity varied between 0.144 and 0.22 m/s due to individual bolus volume variation), providing a shear rate of 71 to 124 s^−1^. Hence a range of shear rates should be expected that governs bolus flow during pharyngeal swallowing. This ranges in most cases is above 50 s^−1^ as is presently mentioned by the National Dysphagia Diet [36]. This is more important in thickened boluses which are non-Newtonian, i.e., the stress (force of deformation) is no longer proportional to the deformation rate (shear rate). Therefore, the shear rate during swallowing is very much individualistic, and is dependent on factors such as an individual’s ability to generate bolus propulsion, individual’s anatomy and pharyngeal geometry.

**Fig. 6:**
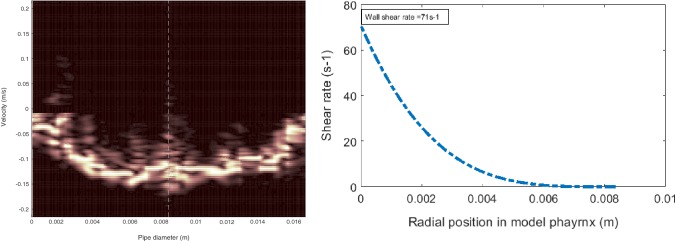
Example of velocity profile (left panel) recorded from the UVP system, for a 15-ml bolus with Fresubin Clear (syrup consistency) over the short axis of elliptical model pharynx providing maximum shear rate of 70 s^−1^ at the wall (right panel). Note in the left panel, power spectra of the whole velocity profile across the geometry are and in right panel, shear rate distribution up to center is shown

Figure 6 presents an example of a typical thickened bolus (Fresubin Clear). In the figure, the velocity profile and corresponding shear rate distribution across radial position, captured with the UVP system is displayed. The maximum bolus velocity recorded was 0.144 m/s that provided a corresponding wall shear rate of 71 s^−1^. The velocity profile in Fig. 6 measured on bolus resembles the one measured in continuous flow (Fig. 5c, d), which validates the result in bolus flow.

The maximum bolus velocity recorded was 0.22 m/s which is in close proximity with the ones from clinical studies by Clavé et al. [37] and Zhu et al. [38], while studying rheology of bolus and swallowing. The velocity in pharyngeal swallow was reported to be 0.31 m/s by Clavé et al. while the one from Zhu et al. were around 0.2 to 0.3 m/s in the hypopharynx region. In general, the literature published suggests bolus velocity varies between 0.1 and 0.5 m/s. Higher value are reported for water and the velocity decreases with increasing bolus consistency. Therefore, the bolus velocity shown in Fig. 6 is considered to be within an acceptable range.

The effect of volume, viscosity, temperature, flow rate, elasticity, gelation properties are beyond the scope of the current work and a separate publication is intended to thoroughly investigate these variables.

### In Vitro Manometry in Continuous Flow

The manometry data for the test fluids are presented in Table 4. The installed pressure transducer showed the differences in pressure applied by the two fluids pumped at two different flow rates: 2.32 and 4.67 l/min (Newtonian fluid oil), 0.70 and 2.34 l/min (non-Newtonian fluid, Fresubin Clear).

**Table 4:** Flow rates, ratio of pressure, high (*P*_H_) and low (*P*_L_), pressure drop (Δ*P*) with respect to the environment, percent difference, pressure drop between pressure and flow rates of the test fluids

Fluids	*Q* (l/min)	(*P*_H_ − *P*_0_)/(*P*_L_ − *P*_0_)	(*Q*_H_/*Q*_L_)^*n*^	% Difference
Rapeseed oil	2.32 and 4.67	2.23	2.03	8.98
Fresubin Clear	0.70 and 2.34	1.39	1.25	9.97

All the numerical values in the table are mean of at least three measurements with standard deviation < 5%

To fulfill the Hagen–Poiseuille relationship mentioned in Eq. 1. Table 4 shows that there was a small difference (< 10%) between rapeseed oil (8.98%) and Fresubin Clear (9.97%) for the two terms, probably because the elliptical geometry induced a secondary flow in the elliptical plane. Furthermore, a contraction of the UES valve may have caused flow irregularities. In summary, it is concluded that reliable pressure data can be acquired from the pressure transducer installed in the model pharynx.

### In Vitro Manometry on Bolus

**Fig. 7:**
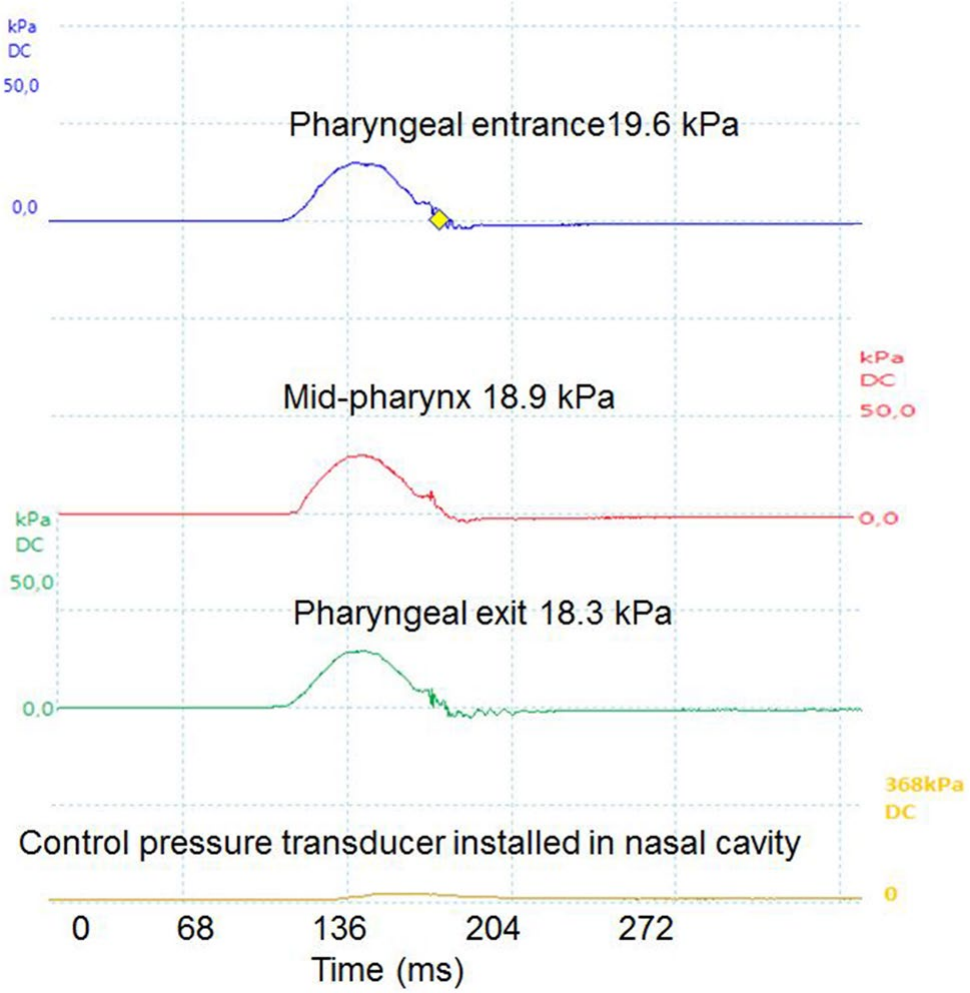
Manometry recorded in the model pharynx on the given four transducers installed. *X*-axis represents time (ms) and *Y*-axis represents pressure range (kPa) on each location in model pharynx. Distortion at the peak is due to mechanical movement. *X*-axis is retyped in Matlab to assist readers. *Note* Control pressure transducer measures atmospheric pressure and is not coming in contact with bolus. Pressure recorded here is subtracted for air pressure correction

To validate that in vitro manometry on bolus in the model, Fig. 7 represents one example of pressure values measured on 15 ml bolus. Slightly higher pressure values were recorded at the pharyngeal entrance 23.38 ± 5.58 kPa, which indicated the flow is pressure driven, followed by the pharyngeal exit (22.93 ± 5.78 kPa) and mid-pharynx (21.84 ± 5.50). The difference in pressure values were not significant (*P* = 0.05) among any of the three locations in the model pharynx. The acquired manometry results (21.84–23.38 kPa), when compared with in vivo results agreed well, indicating the in vitro model is capable of mimicking in vivo type of experiments with respect to different bolus rheological properties, although the in vitro geometry used in the current work is composed of rigid body.

Lin et al. [39, 40] reported maximum pharyngeal pressure of 194.92 mmHg (~ 26 kPa) and 180.9 mmHg (24.12 kPa) in two different studies on thick liquid consistency, while another study by Butler et al. [41] noted maximum pressure values of 100 to 150 mmHg (~ 13–20 kPa) in a honey consistency bolus, as was used here. In both studies however, a volume of 10 ml was used, as compare to 15 ml in the current work.

Our overall conclusion is that pressure transducers perform manometry on bolus in similar way as in clinical examination.

## Conclusion

We have constructed an in vitro model that simulates a part of the human swallowing tract, the pharynx, during bolus passage. The in vitro model encompasses pressure measurements and captures the simultaneous velocity profiles during fluid flow. Validation of the ultrasonic velocity profiles was performed in addition to validation of the pressure measurement during continuous flow. Shear rate measurement on bolus was performed (~ 80 s^−1^ noted here) using ultrasonics and non-invasive in vitro manometry using pressure transducers was performed for the first time here.
